# Biochemistry and Molecular Basis of Intracellular Flavonoid Transport in Plants

**DOI:** 10.3390/plants11070963

**Published:** 2022-04-01

**Authors:** Boas Pucker, Dirk Selmar

**Affiliations:** 1 Institute of Plant Biology, TU Braunschweig, 38106 Braunschweig, Germany; d.selmar@tu-bs.de; 2 Braunschweig Integrated Centre of Systems Biology (BRICS), TU Braunschweig, 38106 Braunschweig, Germany

**Keywords:** anthocyanins, proanthocyanidins, flavonols, flavones, flavonoid transport, flavonoid biosynthesis, flavonoid accumulation, ligandin, MATE, ABCC

## Abstract

Flavonoids are a biochemically diverse group of specialized metabolites in plants that are derived from phenylalanine. While the biosynthesis of the flavonoid aglycone is highly conserved across species and well characterized, numerous species-specific decoration steps and their relevance remained largely unexplored. The flavonoid biosynthesis takes place at the cytosolic side of the endoplasmatic reticulum (ER), but accumulation of various flavonoids was observed in the central vacuole. A universal explanation for the subcellular transport of flavonoids has eluded researchers for decades. Current knowledge suggests that a glutathione S-transferase-like protein (ligandin) protects anthocyanins and potentially proanthocyanidin precursors during the transport to the central vacuole. ABCC transporters and to a lower extend MATE transporters sequester anthocyanins into the vacuole. Glycosides of specific proanthocyanidin precursors are sequestered through MATE transporters. A P-ATPase in the tonoplast and potentially other proteins generate the proton gradient that is required for the MATE-mediated antiport. Vesicle-mediated transport of flavonoids from the ER to the vacuole is considered as an alternative or additional route.

## 1. Introduction

### 1.1. Biological Relevance of Specialized Metabolites

Plants produce an amazing diversity of specialized metabolites to cope with environmental conditions. These compounds are not required for the immediate survival, but provide an evolutionary advantage and are often restricted to particular evolutionary lineages. The various groups of plant metabolites and evolutionary steps towards this diversity were previously reviewed [1,2]. Estimates go up to one million different compounds in the plant kingdom [3], with several thousand being produced by each individual plant [4]. Abiotic stresses like drought, heat, cold, ultra-violet radiation, high light intensities, specific ion concentrations in the soil and many more factors activate biosynthetic pathways. Biotic factors like pathogens and herbivores can also trigger the biosynthesis of specialized defense compounds. Responses to both types of stresses are not mutually exclusive. The flavonoid biosynthesis emerges as an almost universal stress response pathways that is triggered by a broad range of stress conditions [5,6,7,8,9,10,11]. Flavonoids can be classified into several subgroups including flavonols, flavones, anthocyanins, and proanthocyanins [12]. These compounds are synthesized by different branches of the flavonoid biosynthesis [12,13,14,15]. The products of separate branches differ in their biochemical properties thus it can be assumed that they fulfil different biological functions in a plant. Anthocyanins are colorful pigments that are involved in reproduction by attracting animals for pollination and seed dispersal, but they are also significant as stress responses [12,16,17]. Flavonols occur in a wide range of plant parts and are considered as an evolutionary old branch of the flavonoid biosynthesis [18]. They are often produced in response to UV light suggesting an important function in response to this stress [19,20]. Proanthocyanidins (condensed tannins) are colorless polymers of catechin and epicatechin, which turn brown upon oxidation [21]. Functions of proanthocyanidins include protection against reactive oxygen species (ROS) under abiotic stress conditions and protection against herbivores and pathogens [22].

### 1.2. Biosynthesis of Flavonoids

**Figure 1:**
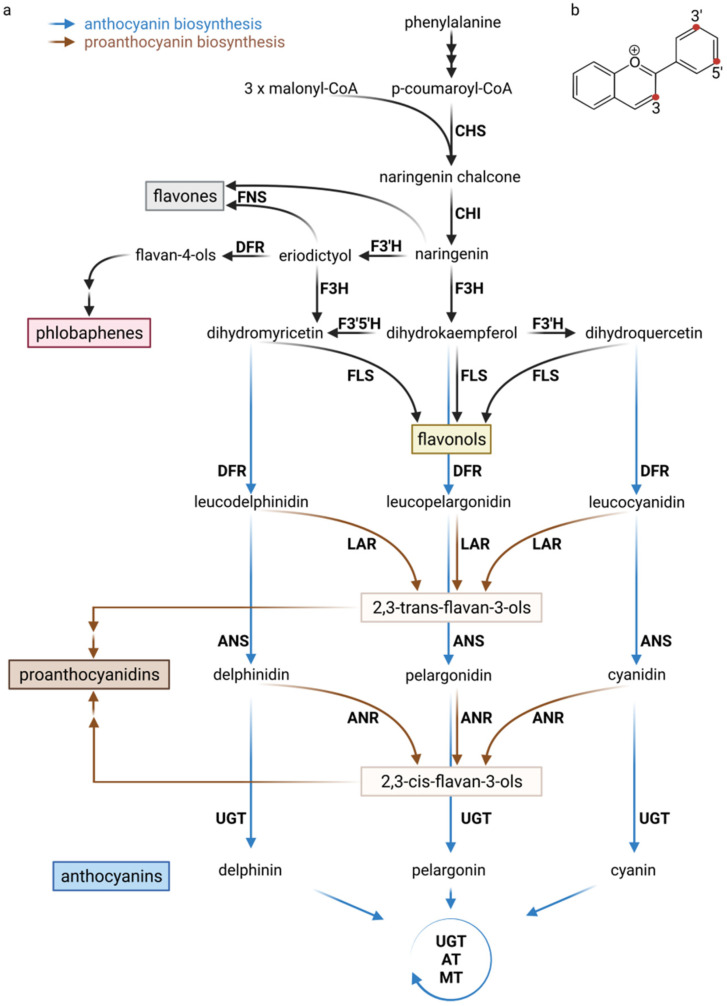
(**a**) Simplified illustration of the flavonoid biosynthesis. CHS (chalcone synthase), CHI (chalcone isomerase), FNS (flavone synthase), FLS (flavonol synthase), F3H (flavanone 3-hydroxylase), F3′H (flavonoid 3′-hydroxylase), F3′5′H (flavonoid 3′5′-hydroxylase), DFR (dihydroflavonol 4-reductase), ANS (anthocyanidin synthase), LAR (leucoanthocyanidin reductase), ANR (anthocyanidin reductase), UGT (UDP-dependent glycosyltransferase), AT (BAHD acyltransferase), and MT (methyltransferase). The successive decoration of anthocyanins with sugar moieties, acyl groups, and methyl groups is indicated by a circle with the names of the responsible enzymes. (**b**) Chemical structure of a flavylium sekeleton of anthocyanidins. Frequently modified positions are highlighted with red dots.

The core pathway of the flavonoid biosynthesis is well conserved and a model system for the specialized plant metabolism (Figure 1), but many unexplored species-specific differences might exist. Briefly, chalcone synthase (CHS) is the first committed enzyme that catalyzes the formation of naringenin chalcone from 4-coumaroyl-CoA and malonyl-CoA [23]. The next step is controlled by the chalcone isomerase (CHI) that isomerizes naringenin chalcone to naringenin [24]. The conversion of naringenin into dihydroflavonol is catalyzed by the flavanone 3-hydroxylase (F3H) [25]. Naringenin can also be channeled into the flavone biosynthesis through the flavone synthase (FNS) [26,27]. Flavonoid 3′-hydroxylase (F3′H) and flavonoid 3′,5′-hydroxylase (F3′5′H) can add additional hydroxyl groups to dihydroflavonols [28,29]. Dihydroflavonols are converted into flavonols by the flavonol synthase (FLS) [30] or into leucoanthocyanidins by the dihydroflavonol 4-reductase (DFR) [31]. Leucoanthocyanindins can be converted into anthocyanindins by the anthocyanidin synthase (ANS) [32,33] or into catechins by the leucoanthocyanidin reductase (LAR) [34]. Anthocyanidins can be converted into epicatechins by the anthocyanidin reductase (ANR) [35] or undergo modification reactions including glycosylations, acylations, and methylations [36,37,38]. The enzymes involved in some branches of the flavonoid biosynthesis are expected to form a metabolon, i.e., are co-located at the surface of the endoplasmatic reticulum (ER) [39,40]. Membrane-bound cytochrome P450 enzymes like F3′H, F3′5′H, and FNS II are forming the cores of these metabolons and attach these clusters of enzymes to the ER [39,41]. The 3-0-glucosylation is usually the first glycosylation step und turns anthocyanidins into anthocyanins [42]. Additional decorations like sugar moieties or acyl groups also influence the stability of anthocyanins [42,43,44]. For example, the addition of coumaroyl or malonyl groups can enhance the in vivo stability substantially [44]. A wide range of decorations is possible, thus explaining the enormous diversity of anthocyanins and flavonoids in general. Enzymes catalyzing these decoration reactions are usually specific for a certain position of the flavonoid aglycone, but can add a wide range of different sugar moieties—often to flavonoids of different subgroups [45,46,47,48]. Following their synthesis, many specialized metabolites like the anthocyanins require transport into the vacuole for long-term storage [49,50,51]. 

Long-term storage might not be the only reason for vacuolar sequestration of flavonoids. It is also plausible that additional modification steps require the extreme conditions of the vacuolar lumen or that the localization of modifying enzymes in the vacuole requires the import of substrates for modification reactions. There are vacuolar glycosyltransferases and acyltransferases that can further modify flavonoids upon sequestration [37]. These enzymes are different from glycosyltransferases and acyltransferases found in the cytoplasm and belong to different evolutionary lineages.

### 1.3. Transport and Subcellular Localizaiton of Flavonoids

Glycosylated forms of anthocyanins, flavonols, flavones, and proanthocyanidin precursors are transported from the cytoplasm into the central vacuole [50]. The molecular mechanisms underlying the transport or diffusion of these metabolites are under investigation for decades. However, the knowledge remained sparse compared to the detailed insights into the biosynthesis of the flavonoid aglycones. Some flavonoid-transport associated genes were identified in *Arabidopsis thaliana*, *Medicago truncatula*, or *Vitis vinifera*, but no universal explanation of the process was achieved yet [8,52]. Two not mutually exclusive models were proposed to explain the transport of flavonoids: direct transport over the tonoplast or vesicle-mediated transport from the ER [53,54]. Mechanisms of flavonoid transport could dependent on the flavonoid class, the cell type, the developmental stage, and various environmental factors. Observations in *Hordeum vulgare* inspired the hypothesis that transport into the vacuole might be controlled by a component of the flavonoid biosynthesis pathway [55]. The authors noticed reduced transport of saponarin (flavone glycoside) in a *chi* mutant. The existence of such regulatory loops might explain why an efficient flavonoid sequestration into the vacuole is essential for high production in barley [55]. This could motivate research on this topic in other plant species and might turn the flavonoid transport into a promising target for the improvement of crop traits.

Flavonoids have also been reported in the nucleus, chloroplasts, and mitochondria [56,57,58,59,60,61,62]. The functions of flavonoids in these compartments remain largely unexplored. One hypothesis suggests that flavonoids in the nucleus protect the DNA [60,63]. Flavonoids might have additional functions in signaling processes and could influence the gene expression [64]. As the biosynthesis enzymes CHS and CHI were also detected in the nucleus, these proteins might be involved in transcriptional regulation or could be responsible for the differential accumulation of flavonoids in nucleus and other compartments [65]. Flavonoids in chloroplasts and mitochondria could have functions in the prevention of reactive oxygen (ROS) formation and ROS scavenging [66].

### 1.4. Membrane Permeability of Specialized Metabolites

A sound comprehension of processes relevant for the transport of flavonoids and other specialized metabolites also requires profound knowledge and consideration of basic physico-chemical coherences. It is beyond debate that any transport of substances within the plant—either from cell to cell or long-distance translocation—requires the transfer of the substance across biomembranes. Biologists have internalized that biomembranes represent efficient borderlines between the different cell compartments. In consequence, it seems to be inevitable that any membrane passage requires the involvement of a corresponding transporter, or carrier systems, respectively. Indeed, this deduction applies to sugars, amino acids and most of the substances involved in primary metabolism. These substances are characterized by a high water solubility. In consequence, they are quite unable to diffuse though the lipophilic zone of bio-membranes. Thus, for their transfer through and across any membrane, transporters are required [67,68,69]. This also applies to ionic nutrients like nitrate, sulphate, or metal ions, whose uptake by the roots necessitates adequate transporters [70,71,72]. By contrast, a tremendous high number of specialized metabolites, i.e., alkaloids, phenolic compounds like flavonoids, or terpenoids, indeed are able to diffuse passively though biomembranes [73]. The comprehensive knowledge about membrane permeability of multifarious substances, and how this ability can be estimated or evaluated, respectively, is premised on extensive studies on the uptake of xenobiotics from the soil [74,75]. Due to their partially hydrophobic and hydrophilic character, most of these substances can diffuse passively through membranes [76,77,78]. According to these insights and coherences, the most important property that enables a substance to simply diffuse through biomembranes is a balanced proportion of hydrophilicity and lipophilicity. This feature is characterized in good approximation by the distribution coefficient of a certain substance in an “octanole-water-system”, i.e., the so-called k_ow_-value, or its decadal logarithm, the log*k*_ow_, respectively, which is also frequently denoted as log *p*-value [79]. It is understood that all substances revealing log *p*-values between −1 and 3 do diffuse through biomembranes [75,80,81]. Indeed, when this realization had been used to predict the passive uptake of pharmaceutical drug, it turned out that some further cognitions are required, and some additional aspects have to be considered for a proper and sound specification of membrane diffusibility of a certain substance. These reflections lead to the argumentation of the “rule of five”, an implementation to predict the membrane permeability that—in addition to the log *p*-value—also considers the size of the molecules, the ability to generate hydrogen bonds, and to act as proton acceptor or donor [82,83]. It is self-evident that these deductions do not only apply to xenobiotics and pharmaceuticals, but also to natural products. This expectation was vividly verified by demonstrating the uptake of alkaloids [84] and coumarins by the roots of various acceptor plants [73,85].

In the light of these considerations, many scientific articles on the translocation of specialized metabolites, which non-reflectively state the involvement of certain transporters for the membrane transfer, could hardly be understood. Yet, even without considering the basic physico-chemical coherences mentioned above, just guided by our daily experience from drinking coffee and tea, or from smoking, it is beyond question that alkaloids, such as caffeine or nicotine are taken up promptly by mucous membranes without the involvement of any carrier. Nonetheless, related carriers are described to be relevant for the translocation, e.g., of nicotine in tobacco [86,87,88]. For elucidating this apparent contradiction, it is vital to consider that the physico-chemical properties of alkaloids are massively impacted by the pH: in acidic solutions, i.e., when the pH is quite lower than the p*K*s-value, the alkaloids are protonated and no longer able to diffuse through biomembranes, whereas in neutral to alkaline solutions, the alkaloids are present as free bases [89,90]. These basic coherences of this phenomenon had been vividly described and presented already half a century ago [91] as so-called ion-trap mechanisms: whereas the free bases passively diffuse from the neutral cytosol through the tonoplast, the protonated alkaloids are trapped in the acidic vacuoles. In this context, the occurrence of certain carriers becomes relevant, because any export of the membrane impermeable protonated alkaloids requires the action of a related carrier. These coherences illustratively outline how the milieu is impacting the ability of a certain substance to passively diffuse through a biomembrane and thereby determine whether or not a transporter is involved in related transport processes. 

Based on the coherences outlined above, it becomes obvious that any discourse on translocation of flavonoids has to consider whether or not a certain molecule is able to passively diffuse though a biomembrane or if an appropriate transporter is required. Most of the flavonoid aglycones reveal a log *p* (Table S1) that expound their inherent ability to diffuse passively across biomembranes. Since these compounds do not exhibit features that might restrict this property according to “the rule of five”, we have to assume that these flavonoids reveal steady membrane permeability. In contrast, the situation is quite different when focusing on the wide-spread derivatives of flavonoids (Table S2) and the positively charged anthocyanidins, whose sound log *p*-values are unfortunately hardly available. Due to the great number of hydroxyl-groups and the positive charge, respectively, these compounds are not able to simply diffuse through biomembranes. Thus, their transfer from one compartment into another requires either a carrier-mediated or a vesicle-based transport [50,54,92].

Here, we review the current knowledge about the intracellular transport and accumulation of flavonoids. This includes tonoplast-based transporters and players associated with a vesicle-based transport system. We also summarized the sparse knowledge about mechanisms underlying the long-range transport of flavonoids within a plant. Finally, we outline open questions that can be addressed by recently developed technologies.

## 2. Ligandin and Transporter-Associated Sequestration of Flavonoids into the Vacuole

**Figure 2:**
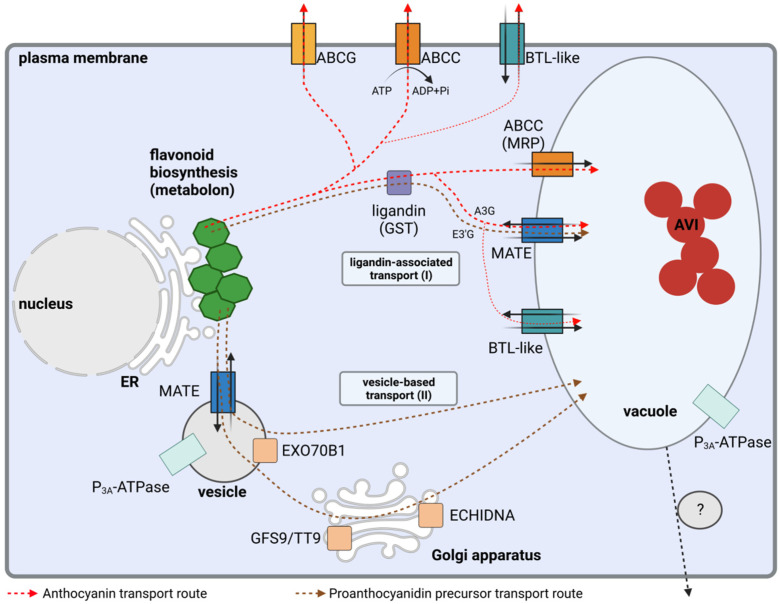
Simplified illustration of the intracellular flavonoid transport pathways. ABCC (ATP-binding cassette (ABC) subfamily C), ABCG (ABC subfamily G), MATE (Multidrug And Toxin Extrusion transporter), BTL-like (bilitranslocase-like), GFS9/TT9 (Green Fluorescent Seed 9/Transparent Testa 9), EXO70B1 (exocyst complex component), ER (Endoplasmatic Reticulum) and AVI (anthocyanin vacuolar inclusion). Strength of lines indicates the assumed relevance of these transport pathways.

Different routes of subcellular flavonoid transport from the ER to the vacuole have been proposed based on numerous observations (Figure 2, Table 1). This involves the movement to the tonoplast and also the crossing of a membrane (tonoplast). One model proposes that vacuolar import of flavonoids is based on a transport system located in the tonoplast [53]. This vacuolar import process involves a ‘ligandin’ [93] that is necessary to escort anthocyanins and precursors of the proanthocyanidins from the ER to the tonoplast where transporters can facilitate the actual uptake. Acidic conditions inside the vacuole induce conformational changes of flavonoids upon import which prevent the flavonoids from crossing a membrane again resulting in retention in the vacuole [94,95].

**Ligandins** are glutathione S-transferase (GST)-like proteins that were reported as a crucial factor for anthocyanin and possibly proanthocyanidin precursor transport in many species: BZ2 in *Zea mays* [96], AN9 in *Petunia hybrida* [97], TT19 in *A. thaliana* [98], PfGST1 in *Perilla frutescens* [99], and VvGST1 and VvGST4 in *Vitis vinifera* [100]. Initially, a detoxification function of these proteins was assumed based on a conjugation with glutathione [101], but it became clear that these proteins are only binding specific flavonoids without catalyzing an enzymatic reaction [102]. It is still an open question whether ligandins have high affinity for anthocyanins or proanthocyanidin (PA) precursors, respectively. Studies investigating the ligandin oder GST homologs of various plant species often complement the *A. thliana tt19* mutant to demonstrate the functionality [51,98,100,103,104,105,106,107,108]. Experiments show that the ligandins of some species only complement the anthocyanin deficit [103,104,105,106], while other studies also observed a restoration of the PA accumulation [51,98,100,107,108]. In summary, these studies suggest that these ligandins of some plant species could be dedicated to the anthocyanin transport. These ligandins could protect the flavonoids, while the actual transport is mediated by membrane proteins. **ATP-binding cassette (ABC) transporters** are a group of primary active transporters, i.e., powered by the consumption of ATP, that were associated with the uptake of flavonoids into the vacuole [50,109,110,111,112]. Many of these flavonoid transporters belong to subgroup C of these ABC transporters and were previously also called multidrug resistance proteins (MRP). Another important group of transporters are the **multidrug and toxin extrusion transporter (MATE)** proteins that are secondary active antiporters, i.e., antiporters that require a proton gradient for the flavonoid import [52,113]. The search for flavonoid transporters was often based on chemical inhibition of specific transporter classes. The primary active ABC transporters are generally inhibited by vanadate, while this does not directly affect antiporters (e.g., MATEs) that take their energy from a proton gradient. In contrast, bafilomycin A1 is an inhibitor of V-type ATPases that disrupts the proton gradient required for secondary active transport. Gramicidin D is an ionophore that also disrupts the proton gradient thus it only effects transporters that rely on this gradient (e.g., MATEs).

Although ABCCs and MATEs were reported in many species this does not rule out the involvement of additional transporters in some species. A gene encoding a protein similar to the secondary active mammalian **bilitranslocase (BTL)** might be involved in the flavonoid transport in *Dianthus caryophyllus* [114] and *Vitis vinifera* [115]. The *D. caryophyllus* protein is inhibited by cyanidin 3-glucoside [114]. The *V. vinifera* protein transports bromosulfalein which is structurally similar to flavonoids [115]. A competitive inhibition of the *V. vinifera* BTL-like protein by quercetin suggests that this is a potential flavonoid transporter [115]. The observation of this protein in berries and the gene expression pattern during the ontogenesis support a potential involvement of the *V. vinifera* candidate in the flavonoid transport [115]. Transport efficiency of a secondary energized transport (proton gradient) is low compared to the directly energized mechanism (ATP consumption) [109]. In summary, this could suggest that BTL is just a minor transport mechanism, while ABCC and MATE in combination with the ligandin could represent the major flavonoid transport mechanism.

**Table 1:** Genes involved in the transport of flavonoids.

Function	Gene	Species	Reference
Ligandin (GST)	*AN9*	*Petunia hybrid (petunia)*	[97]
	*TT19*	*Arabidopsis thaliana*	[98]
	*BZ2*	*Zea mays (maize)*	[96]
	*VvGST1, VvGST4*	*Vitis vinifera (grape vine)*	[100,108]
	*PstGST1*	*Perilla frutescens (deulkkae)*	[99]
	*PpGST1/Riant*	*Prunus persica (peach)*	[116]
	*BnGSTF6, BnGSTF12*	*Brassica napus (rapeseed)*	[117]
	*AcGST1*	*Actinidia chinensis (kiwifruit)*	[107]
	*BRACT1*	*Euphorbia pulcherima (poinsettia)*	[118]
	*RsGST1*	*Raphanus sativus (radish)*	[119]
	*RAP*	*Fragaria vesca (strawberry)*	[103]
	*MdGSTF6*	*Malus domestica (apple)*	[104]
	*LcGST4*	*Litchi chinensis (lychee)*	[120]
	*IbGSTF4*	*Ipomoea batatas (sweet potato)*	[106]
	*CkmGST3*	*Cyclamen spec.*	[121]
	*FL3/DcGSTF2*	*Dianthus caryophyllus (carnation)*	[122]
	*PcGST1*	*Petroselium crispum (parsley)*	[123]
	*CmGST1*	*Chrysanthemum spec.*	[124]
	*CsGSTF1*	*Camelia sinensis (tea)*	[125]
	*DcGST1*	*Daucus carota (carrot)*	[126,127]
	*GmGST26A/GmHsp26A*	*Glycine max (soybean)*	[97]
MATE	*TT12, FFT*	*Arabidopsis thaliana*	[113,128]
	*MtMATE1, MtMATE2*	*Medicago truncatula (barrelclover)*	[52,129]
	*VvAM1, VvAM3*	*Vitis vinifera (grape vine)*	[130,131,132]
	*LhDTX35*	*Lilium spp.*	[133]
	*MdMATE1, MdMATE2*	*Malus domestica (apple)*	[134]
	*BnTT12*	*Brassica napus (rapeseed)*	[135]
	*RsMATE9*	*Raphanus sativus (radish)*	[136]
	*SlMTP77*	*Solanum lycopersicum (tomato)*	[137]
	*VcMATE, 2, 3, 5, 7, 8, 9*	*Vaccinium corymbosum (blueberry)*	[138]
	*GmMATE1*	*Glycine max (soybean)*	[139]
	*FaTT12-1*	*Fragaria vesca (strawberry)*	[140]
	*GhTT12*	*Gossypium hirsutum (cotton)*	[141]
	*DcMATE1*	*Daucus carota (carrot)*	[127]
	*DkMATE1*	*Diospyros kaki (kaki persimmon)*	[142]
ABCC (MRP)	*ZmMRP3(ZmABCC3), ZmMRP4(ZmABCC4)*	*Zea mays (maize)*	[143]
	*AtABCC2*	*Arabidopsis thaliana*	[144]
	*VvABCC1*	*Vitis vinifera (grape vine)*	[145]
	*OsMRP15*	*Oryza sativa (rice)*	[146]
	*RsABC*	*Raphanus sativus (radish)*	[147]
P_3A_-ATPase	*AHA10/TT13*	*Arabidopsis thaliana*	[148,149]
	*PH5*	*Petunia hybrid (petunia)*	[150]
	*GmPH5*	*Glycine max (soybean)*	[151]
H^+^-PPase	*VHP1*	*Arabidopsis thaliana*	[152]
BTL-like	*-* ^1^	*Vitis vinifera (grape vine)*	[115]
	*-* ^1^	*Dianthus caryophyllus (carnation)*	[114]
Vesicle trafficking	*GFS9/TT9*	*Arabidopsis thaliana*	[153]
	*ECHIDNA*	*Arabidopsis thaliana*	[154]
	*EXO70B1*	*Arabidopsis thaliana*	[155]

^1^ Studies were based on antibodies and do not provide gene IDs.

### 2.1. Anthocyanin Transport

The major transporter families ABCC and MATE appear to be involved in anthocyanin transport in *Z. mays* [143], *A. thaliana* [113], *V. vinifera* [130,145], *M. truncatula* [129] and many other species. Primary active ABCC transporters depend on ATP and glutathione for anthocyanin transport, but do not require anthocyanin–glutathione conjugates [102,145]. The *Zea mays* multidrug resistance-associated protein (ZmMRP3) belongs to an ABC transporter subfamily (ABCC) and was identified as a crucial factor for anthocyanin transporter in *Zea mays* [143]. Although ZmMRP3 was necessary for anthocyanin accumulation in the vacuole, experiments with antisense transcripts suggest that an additional transporter is involved in the anthocyanin transport in the aleuron [143]. Based on the expression pattern, it was speculated that *ZmMRP4* could encode an aleuron-specific anthocyanin transporter, but a large deletion renders the resulting protein most likely non-functional and made this look unlikely [143]. The ZmZRP3 ortholog in *A. thaliana*, AtABCC2, is an active ATP consuming transporter required for sequestration of cyanidin 3-O-glucoside, flavone glucosides, and flavonol glucosides into the vacuole [144]. An enrichment of AtABCC2 in the vacuolar membrane fraction suggests that this transporter is located in the tonoplast. Inhibition assays suggest that this ABCC transporter and a H^+^-antiporter work together in the import of flavonoids [144]. This matches a previously proposed hypothesis that suggested that MRP3 might modify the substrate preference of MATE transporters towards anthocyanins [143]. This aligns with reports of the *Medicago truncatula* MATE1 as a high capacity, but low specificity anthocyanin transporter [129] that could require a regulation of its substrate specificity by interaction with an ABCC protein. It seems that ABCC are committed anthocyanin transporters while MATEs are able to transport anthocyanins in addition to other preferred substrates. However, it is surprising that the *AtABBC2* knock-out does not show a flavonoid phenotype [112] and that *AtABBC2* expression is not controlled by the anthocyanin biosynthesis regulators [42]. Nevertheless, the involvement of ABCCs in the transport of anthocyanins is also supported by an analysis of the *V. vinifera* ortholog *VvABCC1* that revealed transport of anthocyanidin 3-O-glucosides and glutathione when heterologously expressed in yeast [145]. A proton gradient over the tonoplast was important for transport of anthocyanins in *V. vinifera* supporting the involvement of MATEs [130]. The proton gradient and vacuole pH are usually controlled by V-ATPases located in the tonoplast, while P-ATPases are located in the plasma membrane. However, the P_3A_-ATPase AHA10/TT13 is involved in the formation of proanthocyanidins and located in the tonoplast [148,149]. The petunia AHA10/TT13 ortholog PH5 was also identified in the tonoplast, where it is hyperactivated by another non-functional transporter [150]. A mutation in the *PH5* gene caused a reduced vacuole acidification in petals that resulted in blue flower color of petunia [150]. This ATPase might be necessary for the secondary active transport of anthocyanins and proanthocyanidins. However, significant *AHA10/TT13* expression was only observed in the seeds of *A. thaliana* and in no other parts of the plant [149], which might indicate that a different mechanism is required to provide the proton gradient for the anthocyanin transport. VPH1 could be a candidate, but it remains unclear whether this weak H^+^-PPase can maintain the proton gradient required for flavonoid transport. An experiment to rescue an *aha10/tt13* mutant through overexpression of *VPH1* resulted only in partial restoration of the wild type phenotype [149].

The existence of several MATE transporter isoforms might be explained by their specificity to certain flavonoid derivatives or their subcellular localization in tonoplast or vesicles, respectively [50,130,132]. Specific additions of methyl and acyl groups could be a regulating factor in the anthocyanin transport [131]. For example, *Medicago truncatula* MATE2 is more affine towards malonylated anthocyanins than towards proanthocyanidin precursors [52]. However, heterologous expression experiments in *A. thaliana* suggest that MtMATE2 might be located in the Golgi and not in the tonoplast [132]. High accumulation of acylated anthocyanins was reported as a likely consequence of overexpression of a specific anthocyanin activating MYB in *A. thaliana* [42] and *Solanum lycopersicum* [156,157]. In summary, ABCC transporters emerged as central for the anthocyanin transports, but MATE transporters could contribute to the process in several species.

### 2.2. Proanthocyanin Transport

Different transporters could be involved in the proanthocyanidin (PA) precursor transport into the vacuole. However, the *A. thaliana* mate/*tt12* mutant shows a lack of proanthocyanidin accumulation [113,158]. At first, AtMATE/AtTT12 appeared to be an anthocyanidin 3-O-glucoside/H^+^-antiporter [113]. Although no transport of glycosylated flavonols, procyanidin dimers, or catechine 3-O-glucoside were observed in vitro, it was proposed that AtMATE/AtTT12 transports glycosylated flavan-3-ols in vivo [113]. A following study demonstrated that AtMATE/AtTT12 transports epicatechin 3′-O-glucoside more effectively than cyanidin 3-O-glucoside [129]. Similar to AtMATE/TT12, a high affinity epicatechin 3′-O-glucoside transporter and a low affinity but high capacity cyanidin 3-O-glucoside transporter was identified in *M. truncatula* hairy root cells, called MtMATE1 [129]. As described for AtMATE/AtTT12 [113], flavonoid aglycones had no inhibitory effect on the transport of the glycosides by MtMATE1, while the two tested glucosides inhibited each others’ transport slightly [129]. *M. truncatula MATE1* is a close homolog of *AtMATE/AtTT12* and was successfully used to complement the *A. thaliana* mate/*tt12* mutant [129]. Several studies provide evidence that plants might modify flavan-3-ols at the 3′-O rather than at the 3-O position, which could explain the observed substrate preferences of AtTT12 [129,159,160].

Many plant species form PAs based on catechins (2,3-trans-flavan-3-ols) and epicatechins (2,3-cis-flavan-3-ols), which are synthesized by leucoanthocyanidin reductase (LAR) and anthocyanidin reductase (ANR), respectively. Due to a lack of LAR activity in *A. thaliana* [34], only the epicatechin pathway is active. It is assumed that glycosylated forms of PA precursors are imported into the vacuole and then condensed into polymers through spontaneous reactions that do not require enzymes [22]. Spontaneous reactions with polysaccharides and other cellular components [161] render PAs insoluble, thus posing a challenge for the experimental investigation of the PA precursor and PA transport. Surprisingly, *aha10/tt13* (ATPase mutant) seeds accumulate more epicatechin than wild type seeds, while the *mate*/*tt12* mutant does not show a difference [113,148]. Additionally, vanillin-reactive PAs were not detectable in the vacuoles of *aha10/tt13* mutants [149]. In summary, MATE transporters were identified as the central transporters of proanthocyanidin precursors, but require a proton gradient generated by an ATPase.

### 2.3. Transport of Other Flavonoids

GSTs can bind flavonol glycosides [102,108] and transport via ABCC transporters was observed in in vitro experiments [144]. ABCC transporters are also responsible for transporting flavones and iso-flavones into the vacuole [144,162,163]. These reports suggest that flavonols and maybe other flavonoids are imported into the vacuole through the same tonoplast-based system as anthocyanins and proanthocyanidin precursors.

## 3. Flavonoid Transport in Vesicles

**Figure 3:**
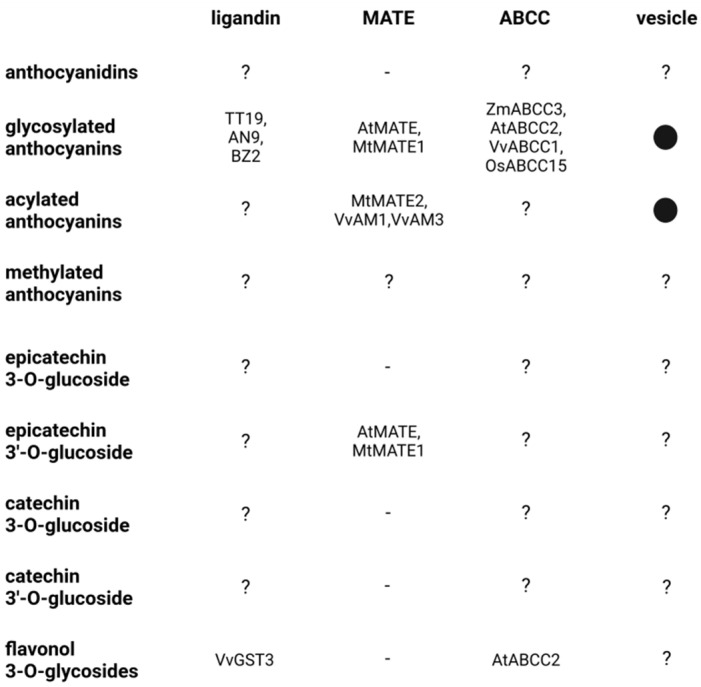
Simplified summary of potential flavonoid transport routes and the involved agents. Example genes are named if the involvement in the transport of the respective compound was reported. Aglycones are not included in this table, because they might be able to pass membranes by diffusion. Vesicle transport is indicated by a dot, the lack of transport ability is indicated by a minus, a lack of knowledge about the transport ability is indicated by a question mark.

There is strong evidence for vesicle-mediated flavonoid transport in many different plant species [49,164,165,166]. Flavonoid-containing vesicles were reported in *Z. mays* [165,167], *Sorghum bicolor* [168], *A. thaliana* [49], and *Ipomoea batatas* [166]. Vesicle transport requires specific tags to ensure that vesicles are delivered to the correct organelle. The required proteins and the implications for the transport of flavonoids have been reviewed previously [8,169] (Figure 3). This vesicle-mediated transport of flavonoids could be an additional or alternative route into the central vacuole. Vesicle transport and direct import into the central vacuole must not be mutually exclusive, because the same mechanisms for transport across the tonoplast could be involved in loading the vesicles [95,129,170,171]. However, it is still debated whether specific components are associated with just one of these transport routes. MATE transporters might be committed to the uptake of flavonoids into the vesicles, but the localization of MATEs in the tonoplast does not allow the exclusion of an involvement in the tonoplast-associated GST/ligandin mechanism in the vesicle mediated flavonoid uptake [50,130]. For example, GST/ligandin could be associated with the direct flavonoid uptake into the vacuole which would require the GST/ligandin to protect anthocyanins during transit through the cytoplasm. However, *A. thaliana tt19* (GST/ligandin) mutants show an enrichment of flavonoid-filled vesicles [172]. This suggests that GST/ligandin is not required for the transfer of flavonoids into the vesicles, but for the unloading of vesicles into the vacuole.

### 3.1. Anthocyanin Transport 

It is assumed that a fusion of anthocyanin-filled vesicles with the tonoplast results in the release of anthocyanins into the central vacuole [49,53,170]. Contradictory microscopic results about the presence/absence of membranes around ‘anthocyanoplasts’ [173] or anthocyanic vacuolar inclusions (AVIs) have been reported in numerous plant species [53,174,175]. It seems that a proteinaceous matrix in the vacuole binds anthocyanins [94,174]. VP24 metalloproteases were repeatedly reported as co-localized with anthocyanins [94,176,177], but the identities of other potentially involved proteins remains an open question. AVIs were reported in different organs and developmental stages including *A. thaliana* seedlings [178], *Dianthus caryophyllus* flowers [174], suspension cell cultures of *Ipomoea batatas* [94], and petals of *Eustonia* spec [164]. However, it remains unclear whether these anthocyanin clusters are surrounded by a membrane [49,131,179] or not [174,180]. A study in *V. vinifera* cell suspension revealed a correlation of anthocyanin content with the formation of AVIs and observed the transport of AVIs from the cytosol into the vacuole [170]. The accumulation of acylated anthocyanins was observed in *V. vinifera* [181] hence AVIs might be a sequestration mechanism for specific types of anthocyanins. AVIs might also be a mechanism to retain anthocyanins in the vacuole as such large anthocyanin clusters are unlikely to be exported easily. Senescence goes along with leakage of membranes and a reduced energy gradient [182] that is required for anthocyanin transport into the vacuole. AVIs might explain how pigments are maintained in the vacuole at this developmental stage [8]. A study in *Zea mays* revealed that vacuolar morphology and AVIs are influenced by light with small vacuoles merging and AVIs releasing anthocyanins into the vacuole upon light exposure [183]. These light induced changes could be responsible for a darkening of the tissue upon light exposure and could be a more general explanation for similar observations in other species [183]. Most epidermal cells of *A. thaliana 5gt* mutants that lack the ability to add sugar moieties at the 5-O position of anthocyanins show the formation of AVIs, while this is rarely the case in the cells of the wild type [178]. A vanadate treatment of seedlings, which inhibits the primary active ABC transporters, resulted in a similar phenotype [178]. The authors present two non-exclusive models to explain these observations: (1) cyanidin 3-O-glucoside could inhibit the breakdown of autophagic bodies which become visible as AVIs and (2) cyanidin 3-O-glucosides and cyanidin 3,5-O-glucosides might be transported by different mechanisms with cyanidin 3-O-glucoside being imported into the vacuole by a vanadate-sensitive transporter and cyanidin 3,5-O-glucoside through a vesicle-based mechanism [178] (vanadate is also inhibiting the ATPases, but firstly those of the plasmalemma). The Golgi-disturbing brefeldin A had no impact on the accumulation of anthocyanins thus indicating that this vesicle transport is trans-Golgi network (TGN)-independent [49]. Anthocyanin-containing sub-vacuolar structures are increased through treatment with vanadate which is a broad range inhibitor of ATPases and ABC transporters. This corroborates the evidence that anthocyanins are accumulating in a sub-vacuolar compartment. Autophagy has been reported as a mechanism that causes the formation of large vesicles from smaller ones [183]. Anthocyanin-filled vesicles in *V. vinifera* hairy roots overexpressing an anthocyanin biosynthesis activating MYB suggest an involvement of vesicles in the anthocyanin sequestration into the vacuole [130,131]. While anthocyanin-transporting MATEs (anthoMATEs) were associated with these vesicles and the tonoplast, GST/ligandin was observed at the presumed ER location [131]. Additional antisense experiments in *V. vinifera* hairy root cells suggest that anthoMATEs and GST/ligandin are involved in different anthocyanin transport mechanisms, because repression of the MATEs resulted in anthocyanin accumulation in the vacuole while repression of the GST resulted in anthocyanin accumulation in vesicles [131]. EXO70B1 is located in vesicles and involved in the internalization of vesicles into the vacuole [155]. The *A. thaliana exo70b1* mutant showed an almost complete loss of anthocyanin pigmentation in the leaves, but the severity of this phenotype decreased during development [155]. This could suggest that only one of the anthocyanin transport routes is affected.

### 3.2. PA Transport

Vesicles directed at the central vacuole and filled with PA precursors have been reported in *A. thaliana* seed coat cells [33,98]. Various *transparent testa* (*tt*) mutants indicate that the lack of seed pigmentation is connected to abnormalities of the vacuole [33,98,148,149]. GREEN FLUORESCENT SEED 9 (GFS9)/TT9 is a protein involved in the intracellular membrane trafficking [153]. The *gfs9/tt9* mutant shows a defect in seed pigmentation, thus it is assumed that this factor is important for the vesicle-based transport of proanthocyanidin precursors. ECHIDNA is another protein associated with the vacuolar trafficking or vacuolar development that is also crucial for the seed pigmentation [154]. Golgi-localized GFS9/TT9 and TGN-localized ECHIDNA are both influencing the seed pigmentation [153,154] supporting the relevance of the vesicle-mediated transport of flavonoids. Since ECHIDNA is required for the trafficking of a TGN-localized vacuolar H^+^-ATPase subunit [184], it is also possible that issues in the protein transport explain the seed color phenotype. An alternative explanation would be that the *gfs9/tt9* or *echidna* mutants disturb the ER organization thus preventing the formation of the flavonoid biosynthesis metabolon [154].

Seeds of the *A. thaliana tt19* (GST/ligandin) mutant revealed an eightfold increased level of insoluble PAs in immature seeds and an absence of epicatechins and their derivatives in the soluble fraction [172]. Moreover, these mutants show an enhanced accumulation of the glycosylated epicatechins, which seem to be the form transported by MATE/TT12 [129,172]. MATE/TT12 can transport PA precursors, but did not show transport of epicatechin aglycons in vitro [113]. The formation of small vesicles filled with PA derivatives in the *tt19* mutant suggests that TT19 is not required for the import into these vesicles, but aberrant PA derivatives might be formed due to the lack of TT19 [172]. In contrast, the accumulation of anthocyanins in *Zea mays* kernels [96] or flavonols in *A. thaliana* pollen grains [185] was not possible without the GST-like protein.

### 3.3. Flavonol Transport

Flavonols were identified in the cytosol instead of the tapetosomes in pollen cells of the tt19 and tt12 mutant [185]. As a consequence, pollen of these mutants were more sensitive to UV radiation with respect to a subsequent germination rate. In contrast to the PAs, flavonols seem to be channeled into the vesicle trafficking system at the ER and not at the vacuole [185]. Generally, the transport of flavonol glycosides is best studied in connection to the seed development. A recent study identified a tapetum-specific flavonol sophoroside transporter (FST1) [186]. The authors demonstrated that this membrane-bound member of the nitrate/peptide transporter family is crucial for the transport and accumulation of flavonol 3-O-sophorosides on the pollen surface.

## 4. Secretion of Flavonoids and Long Distance Transport

For many groups of specialized metabolites it is well-known that the sites of their synthesis and of their accumulation are quite different. Accordingly, these natural products are translocated within the plants, e.g., pyrrolizidine alkaloids are transferred from the roots into the shoots [187], cyanogenic glucosides are allocated from seeds into developing young leaves [188], and glucosinolates are transported from the leaves into the seeds [68]. In general, this allocation from *source* to *sink* organs is realized by a phloem-based transport [187,189]. In contrast, nicotine, which is synthesized in the roots of *Nicotiana sylvestris* plants is translocated into the shoots via xylem [190], driven by the transpiration flow. As a result, nicotine is not accumulated in physiological *sinks*, e.g., the developing seeds [191], but in the transpiring leaves [89]. Unfortunately, with respect to flavonoids such comprehensive investigations of *source* to *sink* tissues and the corresponding insights on putative translocation processes are missing so far.

One of the rare hints that flavonoids might be translocated within plants from one organ to another is based on distinct differences in the composition of flavonoids in *Cuscuta* plants parasitizing on various host plants [192]. As the *Cuscuta* plants take up the substances via their haustoria directly from the vascular bundles of the host, it might be assumed that the observed differences in flavonoid-pattern of the *Cuscuta* plants parasitizing on different plants is due to corresponding differences in composition of flavonoids in the vascular tissues of the various hosts. Support for the presence of flavonoids in phloem and xylem stems from gene expression analyses that suggest that flavone biosynthesis might be active in these organs [193,194]. The substances taken up via the *Cuscuta* haustoria could be derived from both, xylem and phloem [195]. These findings do not give a clue with respect to the localization of the flavonoids in the vascular system of the hosts. Moreover, these findings are not solid proof, since a biosynthesis of the flavonoids by the *Cuscuta* plants themselves could not be fully ruled out [192].

Intercellular flavonoid transport might explain coloration patterns observed in the leaves and flowers of many plant species and could also serve as a stress response. For example, anthocyanins are transported in vascular bundles towards the root tip [196]. The GST-like ligandin might be involved in long-range transport of flavonoids, because it is expressed in the mid vein of leaves in *A. thaliana* showing an expression pattern similar to that of a flavonoid glycosyltransferase [197]. MtMATE2 [52] and RsMATE5 [136] might be involved in long-distance transport of anthocyanins. An ABC transporter that can export genistein and daidzein from the cell was studied in *Glycine max* (soybean) [198]. ABC transporters might transport flavonoids outside the cell, because no glycosylation or acylation is required for transport [50]. Mechanisms to export epicatechin or PA oligomers out of the cell remain unknown. Burst of vacuoles upon cell death is one hypothesis that could explain the PA release from cells [199].

Flavonoid transport between different parts of the plant would be required if biosynthesis could not take place at the target site. Since the precursors of the flavonoid biosynthesis are ubiquitous within a plant, it is likely that most cells would be able to produce flavonoids. Consequently, long-range transport might not be a particularly important mechanism. In summary, more research is required to assess the relevance of intercellular flavonoid transport and to elucidate the molecular mechanisms.

## 5. Conclusions and Open Questions

While the biosynthesis of the flavonoid aglycons is well understood, many questions remain around their modification, intracellular transport, storage, and degradation. How are specific modifications influencing or even controlling the transport? Is controlled transport necessary to achieve the right concentrations in different subcellular compartments, i.e., low concentrations of aglycone products in the cytoplasm and high concentrations of substrates for following reactions in the vacuole? What is the biological relevance of flavonoids in the nucleus, chloroplasts, and mitochondria? Various *transparent testa* (*tt*) mutants do not show complete lack of PAs. Are these observations the results of diffusion across the membrane? Can different anthocyanin biosynthesis activating transcription factors selectively activate specific uptake mechanisms? Additional work on ligandins and vesicle transport could help to achieve a more controlled anthocyanin and PA accumulation in crops. Engineering the flavonoid transport, a potential switch between two competing pathways, could help to increase the nutritional value or the pathogen tolerance of crops. A better understanding could also facilitate the development of ornamental plants with novel pigmentation patterns.

Many transport mechanisms were only observed in a single species or in a small number of species. Results of different studies seem to contradict each other. Systematic comparative studies could provide additional support for these observations and the resulting hypotheses. Since some of the transport mechanisms appear to be specific to certain cell types, the rapid progress in single cell RNA-seq could help to better understand the activity of different players in this process via high-throughput analyses. This technology could enable experiments that distinguish the pathways leading to anthocyanin and proanthocyanin accumulation, respectively. These differences between cell types also emphasize the importance of precise information about the studied material to allow validation by others.

## Supplementary Materials

The following are available online at https://www.mdpi.com/article/10.3390/plants11070963/s1, Table S1: Log *p*-values of various flavonoids. Table S2: Log *p*-values of various flavonoid-glycosides.
