# Supplementary material for: Biochemistry and Molecular Basis of Intracellular Flavonoid Transport in Plants
Source: Plants (Basel). 2022 Apr 1;11(7):963. doi: 10.3390/plants11070963 (PMC9002769; doi:10.3390/plants11070963)
Supplement: Supplementary file 1 [file plants-11-00963-s001.zip › plants-1651573-supplementary.pdf]

Supplemental data

Table S1: Log*P*-values of various flavonoids

	Tsopelas et al.			Rothwell	Dai	Toxnet	ChemSpider		PubChem	∅
	cal.	exp.1	exp.2				cal.	exp.		
Acacetin	3.20	2.56	2.71			3.41	3.14	1.46	2.1	**2.65**
Apigenin	1.74	2.05	2.28	2.92		3.02	2.10	1.14	1.7	**2.12**
Baicalein	3.04	1.94	2.65		3.19		3.31	2.63	1.7	**2.64**
Biochanin A (Olmelin)	3.07	2.75	3.48				3.14	1.36	3.0	**2.80**
Catechin				0.41[1)]			0.49	0.85	0.4	**0.54**
Chrysin	3.52	2.53	3.01			3.52	2.88	1.85	2.1	**2.77**
3´,4´-Dihydroxyflavone	2.64	2.60	2.72				3.08	1.86	2.8	**2.62**
2´,3´-Dihydroxyflavone	2.87	2.59	3.20				3.40		2.8	**2.97**
Daidzein	2.91	2.56	3.10	2.51		2.55	2.78	1.12	2.5	**2.50**
(−)-Epicatechin	0.51	1.63	2.05				0.49	0.85	0.4	**0.99**
Eriodictyol	2.02	2.20	3.02			2.02	2.59	1.14	2.0	**2.14**
Formononetin	2.58	2.04	2.39			3.11	2.96	1.44	2.8	**2.47**
Galangin	2.85	2.55	2.77			2.44	2.83	2.20	2.3	**2.56**
Glycetein				1.97			2.57	1.36	2.4	**2.08**
Genistein	2.57	2.18	2.87	3.04		2.8	2.57	1.36	2.4	**2.47**
Hesperetin	2.60	2.92	3.17			2.60	2.90	1.03	2.4	**2.52**
Hispidulin	2.49	2.68	2.74				1.60	1.38	1.7	**1.98**
Isoliquiritigenin	2.96	2.29	2.95		3.04		3.11	1.67	3.2	**2.75**
Isorhamnetin	2.16	1.81	2.49				1.76	1.73	1.9	**1.98**
Kaempferide	2.82	2.53	2.81				2.74	1.81	2.2	**2.49**
Kaempferol	2.45	2.37	2.44			1.96	2.05	1.49	1.9	**2.09**
Luteolin	2.53	2.22	3.09	3.22		2,53	2.40	1.49	1.4	**2.36**
Liquiritigenin					2.79		2.76	0.87	2.2	**2.16**
Morin	1.54	1.75	1.96			1.54	1.61	1.40	1.5	**1.61**
Myricetin	1.63	1.98	1.84			1.42	2.11	2.18	1.2	**1.89**
Naringenin	2.52	2.97	2.98	2.60		2.52	3.19	0.79	2.4	**2.50**
Quercetin	2.06	2.21	2.45	1.82		1.48		1.37	1.5	**1.84**
Silibinin (Silybin)	2.06	2.31	2.87				2.59	0.86	2.59	**2.21**
Tamarixetin	2.18	2.03	2.52				2.42	1.73	1.9	**2.13**
Tangeretin	2.87	2.87	3.01				2.66	2.58	3.00	**2.83**
Wogonin	2.80	2.62	2.83		3.43		2.14	2.09	3.00	**2.70**

Compilation of log*P*-values from Tsopelas et al. (2017), Rothwell et al. (2005), and Dai et al. (2008) as well as from the databases Toxnet, PubChem (National Library of Medicine) and ChemSpider (Royal Society of Chemistry). Tsopelas et al. (2017) presented data calculated (cal.) using ADME Boxes v. 3.0 software (Pharma Algorithms), as well as experimental data obtained by applying the compounds to two different immobilized artificial membranes (exp.1 and exp.2). In the same manner, at ChemSpider calculated (cal.) as well as experimental log*P*-values are listed. [1)] This log*P* is taken from Poaty et al., 2009. ∅: arithmetic average of all logP-values listed for a certain substance.

Table S2: Log*P*-values of various flavonoid-glycosides

	Tsopelas et al.			Rothwell	Dai	Toxnet	ChemSpider		PubChem	∅
	cal.	exp.1	exp.2				cal.	exp.		
Vitexin 2-O-rhamnoside (Apigenin-8-C-glucoside)	-0.09	1.17	1.45				(1.86)	-1.54	-0.9	**0.02**
Baicalein-7-glucuronide (Baicalin)	0.62	1.34	1.57		1.28		0.31	1.05	1.1	**1.03**
Daidzein-7-glucoside (Daidzin)	0.44	1.37	1.94		0,32		0.45	-0.18	0.7	**0.72**
Daidzein-8-C-glucoside (Puerarin)	1.34	1.74	1.91				1.95	-0.72	0.0	**1.04**
Genistein-7-glucoside (Genistin)	0.45	1.88	2.07	0.97			0.79	-0.26	0.9	**0.97**
Myricetin-3-rhamnoside (Myricitrin)	0.12	1.54	2.21				1.98	1.15	0.5	**1.25**
Naringenin-7-rhamnosyl-glucoside (Naringin)	-0.44	1.56	1.80			-0.44	(2.73)	-1.35	-0.5	**0.10**
Quercetin-3-glucoside (Isoquercitrin)	-0.46	1.59	1.94			-0.10	1.75	0.10	0.4	**0.75**
Quercetin-3-glucoside (xylo-hexofuranoside)				0,76				0.82	0.4	**0.66**
Quercetin-3-rhamnoside (Quercitrin)	0.43	1.53	2.33				0.21	0.80	0.9	**1.03**
Quercetin-4´-glucoside (Spiraeoside)	-0.39	1.07	1.42				-0.38	0.10	0.40	**0.37**
Rutin (Quercetin-3-rhamnoglucoside)	-0.86	1.10	1.61	-0.64		-2.02	(1.76)	-074	-1.3	**-0.41**
Wogonin-7-glucoside					1.16				1.2	**1.18**

Compilation of log*P*-values from Tsopelas et al. (2017), Rothwell et al. (2005), and Dai et al. (2008) as well as from the databases Toxnet, PubChem (National Library of Medicine) and ChemSpider (Royal Society of Chemistry). Tsopelas et al. (2017) presented data calculated (cal.) using ADME Boxes v. 3.0 software (Pharma Algorithms), as well as experimental data obtained by applying the compounds to two different immobilized artificial membranes (exp.1 and exp.2). In the same manner, at ChemSpider calculated (cal.) as well as experimental log*P*-values are listed. ∅: arithmetic average of all logP-values listed for a certain substance. Three calculated log*P*-values from ChemSpider (in brackets), since their values are massively different to the others.
